# Existe uma Associação entre os Resultados de Desempenho do Teste *Timed Up And Go* e o Consumo de Oxigênio de Pico Medido Diretamente em Pacientes com Doença Cardíaca?

**DOI:** 10.36660/abc.20230832

**Published:** 2024-02-16

**Affiliations:** 1 Universidade Federal do Rio Grande do Sul Hospital de Clínicas de Porto Alegre Porto Alegre RS Brasil Programa de Pós-Graduação em Cardiologia e Ciências Cardiovasculares – Universidade Federal do Rio Grande do Sul, Hospital de Clínicas de Porto Alegre, Porto Alegre, RS – Brasil; 2 Universidade Federal do Rio Grande do Sul Hospital de Clínicas de Porto Alegre Grupo de Pesquisa em Cardiologia do Exercício Porto Alegre RS Brasil Grupo de Pesquisa em Cardiologia do Exercício (CardioEx) – Universidade Federal do Rio Grande do Sul, Hospital de Clínicas de Porto Alegre, Porto Alegre, RS – Brasil

**Keywords:** Doenças Cardiovasculares, Consumo de Oxigênio, Avaliação de Resultados em Cuidados de Saúde, Equilíbrio Postural, Teste Timed Up and Go

A capacidade funcional, a aptidão para realizar atividades diárias de forma independente,^1^ é comumente avaliada por meio de testes padronizados, como o teste de caminhada de 6 minutos,^2^ o teste *Timed Up and Go* (TUGT)^3^ e o teste de sentar e levantar de um minuto.^4^ No entanto, o padrão ouro para avaliação da capacidade funcional ou cardiorrespiratória é o teste cardiopulmonar de exercício (TCPE),^5^ que mede o consumo de oxigênio de pico (VO_2_pico). O TCPE envolve o aumento gradual da intensidade do exercício até a exaustão ou início dos sintomas.

Numerosos estudos demonstram consistentemente uma forte associação inversa entre VO_2_pico e eventos cardiovasculares, mortalidade cardiovascular e mortalidade por todas as causas,^5,6^ sublinhando o papel fundamental da capacidade funcional no contexto das doenças cardiovasculares. Além disso, na insuficiência cardíaca, independentemente do status da fração de ejeção, o mau desempenho no teste de caminhada de 6 minutos tem sido associado a riscos elevados de mortalidade por todas as causas e insuficiência cardíaca.^7^

Nesta edição dos Arquivos Brasileiros de Cardiologia, Santos et al.^8^ exploraram a relação entre o TUGT, uma medida do tempo que uma pessoa leva para se levantar de uma cadeira, caminhar uma distância de 3 metros e depois sentar-se novamente, e VO_2_pico em indivíduos com insuficiência cardíaca ou doença arterial coronariana. O estudo incluiu 200 participantes (com idades entre 36 e 92 anos; 70% do sexo masculino) inscritos em um programa de reabilitação cardíaca. Notavelmente, 30% dos participantes tiveram insuficiência cardíaca, enquanto 70% tiveram doença arterial coronariana. Todos os participantes foram submetidos ao TUGT e ao TCPE para avaliar sua capacidade funcional. Os pesquisadores desenvolveram uma equação baseada no desempenho do TUGT que previu com precisão o VO_2_pico, alcançando uma área sob a curva de 0,80. Foi identificado um ponto de corte do TUGT de 5,47 segundos para predizer VO_2_pico ≥ 20 ml.kg-¹.min-¹, com sensibilidade de 83% e especificidade de 67%.^8^ Esse ponto de corte é clinicamente significativo, pois pode ser utilizado para estratificar o risco em pacientes com insuficiência cardíaca. Indivíduos com VO_2_pico> 20 ml.kg-¹.min-¹ apresentam baixo risco, garantindo mais de 95% de sobrevida livre de eventos em 1 ano. Por outro lado, aqueles com VO_2_pico < 14 ml.kg-¹.min-¹ enfrentam um risco de mortalidade superior a 20% no mesmo período de um ano.^9^

A capacidade preditiva do TUGT para VO_2_pico tem implicações clínicas significativas, oferecendo aos profissionais de saúde um meio conveniente e eficiente para avaliar a capacidade funcional de um indivíduo e, posteriormente, o seu risco de resultados cardiovasculares adversos. Além disso, o TUGT vai além do seu papel como preditor do VO_2_pico, servindo como uma ferramenta valiosa para avaliar a funcionalidade global. Ao quantificar o tempo necessário para um indivíduo se levantar de uma cadeira, percorrer uma curta distância e depois retornar à posição sentada, o TUGT fornece insights sobre a capacidade da pessoa de realizar atividades diárias essenciais. Esta informação é fundamental para o planejamento da reabilitação, monitoramento do paciente e avaliação abrangente da qualidade de vida geral.

Apesar da hipótese convincente apresentada pelos autores, é crucial reconhecer as limitações inerentes ao estudo. Sua validade externa é limitada pelo seu desenho unicêntrico no Brasil e pelo tamanho amostral relativamente pequeno. Além disso, a ausência de informações sobre possíveis variações nos procedimentos de teste entre os grupos de insuficiência cardíaca e doença arterial coronariana introduz uma camada adicional de complexidade. O perfil étnico dos participantes (brancos, negros etc.) e o seu potencial impacto nos resultados também permanece pouco claro. Notavelmente, a maioria dos pacientes deste estudo pertencia às classes funcionais I e II da NYHA, com apenas 10% enquadrando-se nas classes III e IV. Portanto, deve-se ter cautela ao extrapolar esses achados para indivíduos com insuficiência cardíaca, particularmente aqueles com capacidade física mais limitada. Finalmente, embora elogiemos os esforços dos autores, é necessária uma avaliação abrangente e validação das associações entre o teste TUGT e o VO_2_pico medido diretamente. Investigações em larga escala, abrangendo um espectro mais amplo de participantes, são indispensáveis.
