# Supplementary material for: Existe uma Associação entre os Resultados de Desempenho do Teste Timed Up And Go e o Consumo de Oxigênio de Pico Medido Diretamente em Pacientes com Doença Cardíaca?
Source: Arq Bras Cardiol. 2024 Feb 16;120(12):e20230832. [Article in Portuguese] doi: 10.36660/abc.20230832 (PMC11021033; doi:10.36660/abc.20230832)
Supplement: Translation

# Translation: Is There an Association Between Timed Up and Go Test Performance Results and Directly Measured Peak Oxygen Consumption in Patients with Heart Disease?

**Affiliations:** 1 Porto Alegre RS Brazil Programa de Pós-Graduação em Cardiologia e Ciências Cardiovasculares – Universidade Federal do Rio Grande do Sul, Hospital de Clínicas de Porto Alegre, Porto Alegre, RS – Brazil; 2 Porto Alegre RS Brazil Grupo de Pesquisa em Cardiologia do Exercício (CardioEx) – Universidade Federal do Rio Grande do Sul, Hospital de Clínicas de Porto Alegre, Porto Alegre, RS – Brazil

**Keywords:** Cardiovascular Diseases, Oxygen Consumption, Outcome Assessment, Health Care, Postural Balance, Test Timed Up and Go

Functional capacity, the ability to perform daily activities independently,^1^ is commonly assessed using standardized tests like the 6-minute walk test,^2^ Timed Up and Go Test (TUGT),^3^ and one-minute sit-to-stand test.^4^ However, the gold standard for evaluating functional or cardiorespiratory capacity is cardiopulmonary exercise testing (CPET),^5^ which measures peak oxygen consumption (VO_2_peak). CPET involves gradually increasing exercise intensity until exhaustion or symptom onset.

Numerous studies consistently demonstrate a strong inverse association between VO_2_peak and cardiovascular events, cardiovascular mortality, and all-cause mortality,^5,6^ underscoring the pivotal role of functional capacity in the context of cardiovascular diseases. Moreover, in heart failure, regardless of ejection fraction status, poor performance on the 6-minute walk test has been linked to elevated risks of all-cause mortality and heart failure.^7^

In this issue of the *Arquivos Brasileiros de Cardiologia*, Santos et al.^8^ explored the relationship between the TUGT, a measure of the time it takes for a person to rise from a chair, walk a distance of 3 meters and then sit down again, and VO_2_peak in individuals with heart failure or coronary artery disease. The study included 200 participants (aged 36 to 92 years; 70% males) enrolled in a cardiac rehabilitation program. Remarkably, 30% of the participants had heart failure, while 70% had coronary artery disease. All participants underwent both TUGT and CPET to evaluate their functional capacity. The researchers devised an equation based on TUGT performance that accurately predicted VO_2_peak, achieving an area under the curve of 0.80. A TUGT cutoff point of 5.47 seconds was identified to predict a VO_2_peak ≥ 20 ml.kg-¹.min-¹, with a sensitivity of 83% and a specificity of 67%.^8^ This cutoff point holds clinically significant as it can be utilized to stratify risk in patients with heart failure. Individual with a VO_2_peak > 20 ml.kg-¹.min-¹ exhibit a low risk, ensuring over 95% event-free survival at 1 year. Conversely, those with a VO_2_peak < 14 ml.kg-¹.min-¹ face a greater than 20% mortality risk at the same one-year mark.^9^

The predictive capacity of the TUGT for VO_2_peak has significant clinical implications, offering healthcare providers a convenient and efficient means to evaluate an individual's functional capacity and, subsequently, their risk of adverse cardiovascular outcomes. Moreover, the TUGT goes beyond its role as a predictor of VO_2_peak, serving as a valuable tool for assessing overall functionality. By quantifying the time required for an individual to rise from a chair, cover a short distance, and then return to a seated position, the TUGT provides insights into the person's ability to perform essential daily activities. This information is pivotal for rehabilitation planning, patient monitoring, and comprehensive assessment of overall quality of life.

Despite the compelling hypothesis presented by the authors, it is crucial to acknowledge the inherent limitations of the study. Its external validity is constrained by its single-center design in Brazil and a relatively small sample size. Moreover, the absence of information regarding potential variations in testing procedures between the heart failure and coronary artery disease groups introduces an additional layer of complexity. The ethnic profile of the participants (white, black, etc.) and its potential impact on the results also remains unclear. Notably, the majority of patients in this study belonged to NYHA functional classes I and II, with only 10% falling into classes III and IV. Therefore, caution should be exercised when extrapolating these findings to individuals with heart failure, particularly those with more limited physical capacity. Finally, while we commend the authors’ efforts, a comprehensive assessment and validation of the associations between the TUGT test and directly measured VO_2_peak is necessary. Larger-scale investigations encompassing a broader spectrum of participants are indispensable.
